# Clinical characteristics of diffuse large B-cell lymphoma complicated with multiple primary malignant neoplasms

**DOI:** 10.3389/fonc.2025.1592517

**Published:** 2025-05-21

**Authors:** Mingxi Tian, Xuejiao Gu, Bin Lv, Yanhui Li, Ziqing Huang, Xinyi Li, Yan Zhang, Ying Wang, Feng Zhu

**Affiliations:** Department of Hematology, Affiliated Hospital of Xuzhou Medical University, Xuzhou, Jiangsu, China

**Keywords:** clinical features, diffuse large B-cell lymphoma, multiple primary malignant neoplasms, prognosis, survival analysis

## Abstract

**Objective:**

To investigate the clinical features and prognosis of diffuse large B-cell lymphoma (DLBCL) combined with multiple primary malignant neoplasms (MPMNs).

**Methods:**

The clinical data and prognosis of 31 patients with DLBCL combined with MPMNs diagnosed by pathology between January 2012 and April 2024 in the Department of Hematology, Affiliated Hospital of Xuzhou Medical University were retrospectively analyzed.

**Results:**

Between January 2012 and April 2024, a total of 937 patients were diagnosed with DLBCL, among whom 31 patients had MPMN, with an incidence rate of 3.3%. The cases were divided into two groups according to the different occurrence intervals of the two tumor types. There were 10 patients in the synchronous MPMN group and 21 in the metachronous MPMN group. Statistically significant differences in gender (*P*=0.046), lactate dehydrogenase levels (*P*=0.040), and B symptoms (*P*=0.022) were noted between the two groups. The median age at first (Age 1) and second (Age 2) malignancy diagnoses was 62 (32–87) and 65 (38–87) years, respectively. The median interval between the two tumors was 15 (0–300) months. Kaplan-Meier survival analysis revealed that Age 1≥60 years, synchronous tumors, International Prognostic Index score of medium, high, and high-risk groups (3–5 points), interval time of two malignancies of <50 months, B symptoms, elevated LDH level, Eastern Collaborative Oncology Group score of ≥2 points, and radiotherapy and chemotherapy for the first tumor were adverse factors affecting overall survival.

**Conclusions:**

DLBCL combined with MPMNs is rare in the clinic. Age 1≥60 years and shorter time interval between the two tumors are the main factors affecting poor prognosis. Early diagnosis and treatment of DLBCL with MPMNs should be prioritized clinically to prevent misdiagnosis and enhance patient outcomes.

## 1 Introduction

Lymphoma is a hematological malignancy with an increasing incidence rate. Diffuse large B-cell lymphoma (DLBCL) is the most common lymphoma subtype. Advancements in diagnostic and therapeutic techniques in recent years, particularly the clinical use of rituximab-based immunochemotherapy, have significantly improved the prognosis of DLBCL patients. However, the risk of developing multiple primary malignant neoplasms (MPMNs) has also increased. MPMN refers to the occurrence of two or more primary malignant tumors in the same individual, either simultaneously or sequentially, in the same or different organs or systems (1). Recent reports in China indicate a rising trend in the incidence of MPMNs (2). Patients with MPMNs generally have poor therapeutic outcomes and prognosis, making the study of MPMN risk factors, pathogenesis, and prognosis a key focus of international research. However, large-scale case studies are still lacking. Identifying the clinical features and prognostic factors of DLBCL with MPMNs can improve prognostic assessment and help clinicians develop precise treatment and follow-up strategies. The present study retrospectively analyzed 31 cases of DLBCL with MPMNs in order to provide clinical insights.

## 2 Materials and methods

### 2.1 General information

A retrospective analysis was conducted on the clinical data of 31 patients diagnosed with DLBCL combined with MPMNs and treated in the Department of Hematology, the Affiliated Hospital of Xuzhou Medical University between January 2012 and April 2024. All cases of DLBCL were primary, and any transformed cases were excluded. The diagnosis of MPMN was based on the revised Warren and Gates criteria (1),with comprehensive evaluation of pathological morphology, immunophenotype, anatomical location, and imaging data to exclude metastatic or recurrent tumors. Inclusion criteria were as follows: (1) diagnosis of lymphoma and MPMNs that was confirmed by histopathology, with lymphoma subtypes classified according to the fifth edition of the World Health Organization Classification of Tumors of Hematopoietic and Lymphoid Tissues; (2) presence of complete medical and follow-up records; (3) conduct of necessary pre-treatment evaluations. Exclusion criteria included: (1) inability to confirm whether tumors were primary, metastatic, or recurrent; (2) co-occurrence of more than two tumor types; (3) co-infections with HIV, hepatitis B, or hepatitis C viruses; and (4) presence of incomplete clinical data. All patients provided written informed consent. The study was approved by the ethics committee of the Affiliated Hospital of Xuzhou Medical University and was carried out in accordance with the ethical standards formulated in the Helsinki Declaration.

### 2.2 Diagnostic criteria

Currently, there is no internationally recognized definition or diagnostic standard for MPMNs. The present study adopted the following criteria proposed by Warren and modified by Gates1: (1) all tumors are pathologically diagnosed as malignant; (2) the pathological and morphological features of each tumor are independent of each other; and (3) metastasis, recurrence of cancer, and other conditions are excluded. The diagnosis of lymphoma conforms to the “2021 Chinese Society of Clinical Oncology Lymphoma Diagnosis and Treatment Guidelines.” According to Moertel et al.,3 MPMN is classified into two types based on the interval between the diagnosis of the two tumors. Synchronous MPMN refers to the occurrence of two tumors simultaneously or within six months of each other, while metachronous MPMN refers to the occurrence of two tumors more than six months apart.

### 2.3 Research indicators

(1) Clinical characteristics were as follows: gender, age, Ann Arbor staging, lymphoma classification, presence of B symptoms, Eastern Cooperative Oncology Group (ECOG) score, interval between the onset of DLBCL and MPMN, primary site, number of extranodal involvements, treatment regimen, and survival status. (2) Laboratory indicators included: hemoglobin (HB) level, lactate dehydrogenase (LDH) level, International Prognostic Index (IPI) score, Hans classification, and β_2_-microglobulin (β_2_-MG) level.

### 2.4 Follow-up

Follow-up was conducted through telephone interviews, outpatient visits, or hospital records. The period of follow-up was defined as the time from the diagnosis of the second malignancy. Follow-up content included the patient’s survival and treatment status. Overall survival (OS) was defined as the time from the initial diagnosis of the malignant tumor until death from any cause or the end of the follow-up. The last follow-up time was used as the endpoint for the patients lost to follow-up during the study.

### 2.5 Statistical methods

Statistical analysis was performed using IBM SPSS Statistics (Version 25.0; IBM Corporation, https://www.ibm.com/analytics/spss-statistics-software) and GraphPad Prism (Version 6.0; GraphPad Software, LLC, https://www.graphpad.com/scientific-software/prism/).Continuous data were presented as mean ± standard deviation. The Kaplan-Meier method was used to calculate the median survival time and plot survival curves. The log-rank test was used to compare survival differences between groups. Multivariate analysis was performed using the Cox proportional hazards model to analyze prognostic factors, with *P*<0.05 considered statistically significant. Categorical data were evaluated using the chi-square test. Fisher’s exact test was used when the sample size was <40, the expected frequency in the contingency table was <1, or when more than 1/5 of the expected frequencies were <1.

## 3 Results

### 3.1 Clinical characteristics

The present study included 31 patients diagnosed with DLBCL combined with MPMNs at the Affiliated Hospital of Xuzhou Medical University. The patients were divided into two groups based on the difference in the interval between the occurrence of the two tumors as follows: there were 10 patients in the synchronous MPMN group and 21 patients in the metachronous MPMN group. **Table 1** lists the general patient characteristics. These baseline features are recorded at the time of the first DLBCL diagnosis. There were 19 females (61.3%) and 12 males (38.7%), 90% of patients in the synchronous group were female, compared to 47.6% in the metachronous group. This difference was statistically significant (*P*=0.046). The age at diagnosis of the first malignancy (Age 1) ranged from 32 to 87 years, with a median age of 62 years. The age at second malignancy diagnosis (Age 2) ranged from 38 to 87 years, with a median age of 65 years. The majority of patients were ≥60 years old at the time of diagnosis of both tumors, accounting for 54.8% (17/31) of the study cohort. The median interval from the diagnosis of the first malignancy to the occurrence of the second tumor was 15 months (0–300 months), with 74.2% (23/31) of patients having an interval of <50 months and 25.8% (8/31) having an interval of >50 months. The difference between the two groups was statistically significant (*P*=0.032). The differences in LDH level elevation and presence of B symptoms between the two groups were also statistically significant (*P*=0.040, *P*=0.022, respectively). Among the 31 patients, 14 (45.2%) were in Ann Arbor stage I/II, and 17 (54.8%) were in stage III/IV. According to the patients’ IPI scores, 21 patients were in the low/low-middle risk group (0–2 points), and 10 patients were in the middle-high/high-risk group (3–5 points). There was no statistically significant difference between the two groups in terms of lymphoma staging and IPI scores (*P*=0.280, *P*=0.222, respectively). There was also no statistically significant difference in the interval between the occurrence of the two tumors based on β_2_-MG level, number of extranodal involvements, primary site, Hans classification, HB level, and ECOG score (*P*=0.074, *P*=0.381, *P*=0.247, *P*=0.447, *P*=0.135, and *P*=0.677, respectively).

**Table 1:** Comparison of clinical data between synchronous and metachronous MPMN patients.

Factor	Synchronous Group (n)	Metachronous Group (n)	Total (n)	*P* value
Gender				*0.046
Female	9	10	19	
Male	1	11	12	
Age 1 (years)				0.280
<60	3	11	14	
≥60	7	10	17	
Age 2 (years)				1.000
<60	3	6	9	
≥60	7	15	22	
Stage				0.280
I or II	3	11	14	
III or IV	7	10	17	
IPI				0.222
0–2	5	16	21	
3–5	5	5	10	
LDH				*0.040
High	6	4	10	
Normal	4	17	21	
β_2_-MG				0.074
High	5	3	8	
Normal	5	18	23	
Extranodal involved				0.381
<2	4	4	8	
≥2	6	17	23	
B Symptoms				*0.022
No	5	19	24	
Yes	5	2	7	
Primary Site				0.247
Extranodal	4	14	18	
Nodal	6	7	13	
HB				0.135
<110 g/L	7	8	15	
≥110 g/L	3	13	16	
TF2T (months)				*0.032
<50	10	13	23	
≥50	0	8	8	

MPMN, multiple primary malignant neoplasm; Age 1, age at first malignancy diagnosis; Age 2, age at secondary malignancy diagnosis; IPI, International Prognostic Index; LDH, lactate dehydrogenase; β_2_-MG, β_2_-microglobulin; HB, hemoglobin; TF2T, time free to second tumor. *P<0.05.

### 3.2 Analysis of 21 metachronous MPMN cases

Of the 31 DLBCL combined with MPMN patients, 21 had metachronous dual malignancies, accounting for 67.7% of the cohort (**Table 2**). Among these, 11 were male and 10 were female. Based on the Hans classification, 21 DLBCL combined with MPMN patients were divided into germinal center B-cell-like (GCB) (14 cases, 66.7%) and non-germinal center B-cell-like (NonGCB) (seven cases, 33.3%) cases. DLBCL diagnosis occurred before the other tumor in nine cases, while the other tumor developed before DLBCL in 12 cases (ratio of 3:4). The age at onset of the first malignancy ranged from 32 to 79 years, with a median age of 58 years. The age at the second tumor onset ranged from 38 to 80 years, with a median age of 65 years. The median interval between the first and second malignancy was four years, ranging from 11 to 300 months. Among the 21 patients, one patient (case number 3) developed acute promyelocytic leukemia (APL) after DLBCL diagnosis with an interval of 72 months. The remaining 20 patients developed different types of solid tumors, including: three cases of thyroid cancer, three cases of lung cancer, two cases of gastric cancer, two cases of cervical cancer, two cases of endometrial cancer, two cases of esophageal cancer, and one case each of ovarian cancer, colon cancer, nasopharyngeal cancer, renal cancer, breast cancer, and prostate cancer. Among the 21 patients, 17 received chemotherapy as the main treatment for DLBCL, and only two (9.52%) were treated with combined chemotherapy and radiotherapy. Two patients also received chemotherapy combined with autologous hematopoietic stem cell transplantation (HSCT). The majority of patients chose a surgical approach for the treatment of solid tumors, accounting for 61.90% of the cohort (13/21). Two patients (case numbers 8 and 16) received combined chemotherapy and radiotherapy as a comprehensive treatment (**Table 3**).

**Table 2:** Clinical characteristics of 21 patients with metachronous MPMN.

No.	Gender	Second Cancer Type	Age 1 (years)	Age 2 (years)	TF2T	Hans Type	Survival Status	OS (months)
1	Male	Thyroid Cancer	58	60	26	GCB	Alive	60
2	Female	Cervical Squamous Cancer	54	59	48	GCB	Alive	119
3	Female	APL	32	38	72	GCB	Alive	99
4	Female	Ovarian Serous Adenocarcinoma	74	79	60	NonGCB	Deceased	65
5	Female	Colorectal Adenocarcinoma	76	79	36	GCB	Alive	41
6	Male	Thyroid Cancer	50	62	144	NonGCB	Deceased	185
7	Female	Endometrial Adenocarcinoma	46	50	48	NonGCB	Alive	70
8	Female	Nasopharyngeal Cancer	49	50	12	GCB	Alive	33
9	Male	Thyroid Cancer	58	65	84	GCB	Deceased	102
10	Male	Esophageal Squamous Cancer	65	66	12	NonGCB	Alive	13
11	Male	Gastric Adenocarcinoma	69	79	132	NonGCB	Deceased	141
12	Female	Lung Adenocarcinoma	79	80	11	GCB	Deceased	32
13	Female	Cervical Squamous Cancer	38	63	300	GCB	Alive	313
14	Male	Esophageal Squamous Cancer	69	77	96	GCB	Alive	111
15	Male	Prostate Cancer	69	70	12	GCB	Deceased	34
16	Female	Endometrial Adenocarcinoma	66	67	12	GCB	Deceased	16
17	Male	Lung Adenocarcinoma	62	66	48	GCB	Deceased	62
18	Female	Gastric Adenocarcinoma	56	59	36	NonGCB	Alive	37
19	Male	Small Cell Lung Cancer	60	61	15	NonGCB	Alive	26
20	Male	Renal Cancer	53	56	36	GCB	Alive	46
21	Male	Breast Cancer	57	78	252	GCB	Alive	259

MPMN, multiple primary malignant neoplasm; Age 1, age at first malignancy diagnosis; Age 2, age at secondary malignancy diagnosis; TF2T, time free to second tumor; OS, overall survival; APL, acute promyelocytic leukemia; GCB, germinal center B-cell-like; NonGCB, non-germinal center B-cell-like.

**Table 3:** Treatment conditions of 31 DLBCL patients with MPMN.

Treatment	Synchronous Group		Metachronous Group	
	DLBCL	Second Tumor	DLBCL	Second Tumor
ST	0	4	0	13
CT	8	2	17	6
CT+RT	0	0	2	2
CT+HSCT	1	1	2	0
RT	0	1	0	0
No Treatment	1	2	0	0

DLBCL, diffuse large B-cell lymphoma; MPMN, multiple primary malignant neoplasm; CT, chemotherapy; RT, radiotherapy; ST, surgical treatment; HSCT, hematopoietic stem cell transplantation.

### 3.3 Analysis of 10 synchronous MPMN cases

There were 10 cases of synchronous dual malignancies, accounting for 32.35% of the cohort (**Table 4**). Among these, nine were female and one was male. The age at onset ranged from 38 to 87 years, with a median age of 67 years. Five patients (case numbers 2, 4, 6, 9, and 10) were diagnosed during surgery or routine preoperative examinations, and five patients were diagnosed during follow-up for the primary disease, with all diagnoses occurring within six months of the first malignancy. Four patients were diagnosed with a second tumor within five months of the first malignancy diagnosis, and one patient (case number 8) was diagnosed with the second tumor after four months. There were five cases each of the GCB and NonGCB subtypes based on the Hans classification. Among the 10 patients, two had kidney cancer, two had liver cancer, two had ovarian cancer, and one each had colon cancer, myelodysplastic syndrome (MDS), lung cancer, and cervical cancer. Eight patients received chemotherapy as the main treatment for DLBCL, one patient (case number 7) received combined chemotherapy and HSCT, and one patient (case number 6) did not receive any treatment. Among the 10 patients, four underwent surgical treatment for solid tumors, two received chemotherapy, one (case number 3) received combined chemotherapy and HSCT, one (case number 9) underwent radiotherapy for liver cancer, and two patients (case numbers 4 and 6) did not receive any treatment (**Table 3**).

**Table 4:** Clinical characteristics of 10 patients in synchronous MPMN group.

No.	Gender	Age	Second tumor type	Hans Type	Survival status	OS (months)
1	Female	87	Renal Pelvic Papillary Carcinoma	GCB	Deceased	6
2	Female	69	Colon Adenocarcinoma	NonGCB	Deceased	5
3	Female	38	MDS	NonGCB	Alive	10
4	Female	75	Lung Adenocarcinoma	GCB	Alive	6
5	Female	53	Cervical Squamous Cancer	NonGCB	Deceased	12
6	Male	62	Hepatocellular Carcinoma	NonGCB	Deceased	1
7	Female	53	Ovarian Serous Adenocarcinoma	NonGCB	Alive	18
8	Female	65	Renal Clear Cell Carcinoma	GCB	Deceased	7
9	Female	71	Hepatocellular Carcinoma	GCB	Deceased	3
10	Female	69	Ovarian Cancer	GCB	Alive	8

MPMN, multiple primary malignant neoplasm; OS, overall survival; GCB, germinal center B-cell-like; NonGCB, non-germinal center B-cell-like; MDS, myelodysplastic syndrome.

### 3.4 Survival and prognosis of patients with DLBCL combined with MPMNs

**Figure 1:**
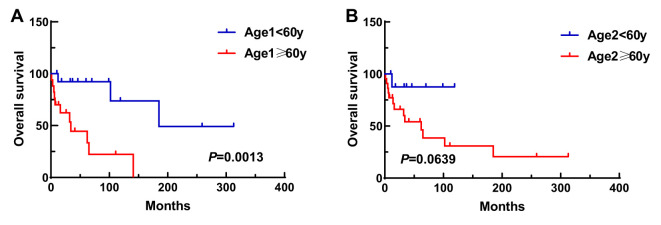
Impact of Age 1 **(A)** and Age 2 **(B)** on patient survival. Age 1, age at first malignancy diagnosis; Age 2, age at secondary malignancy diagnosis.

**Figure 2:**
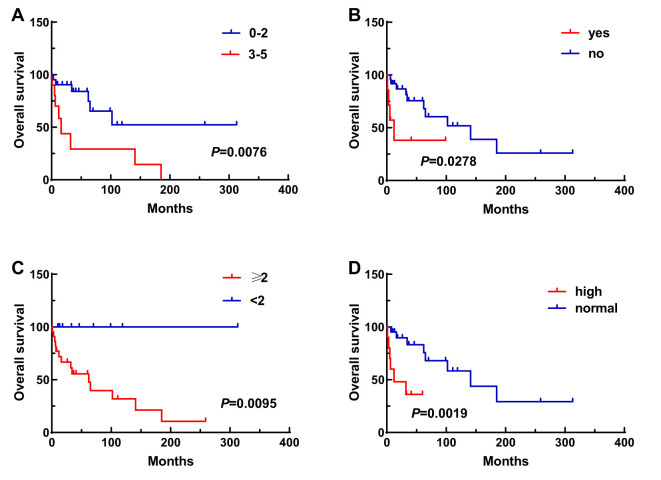
Impact of various clinical factors on patient survival, including IPI score **(A)**, B symptoms **(B)**, ECOG score **(C)**, and LDH levels **(D)**.

**Figure 3:**
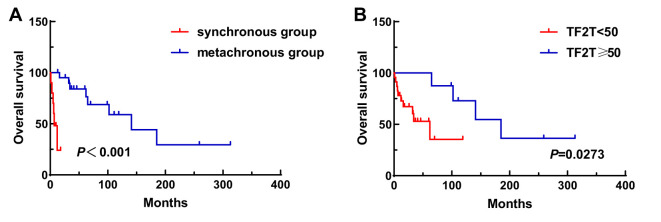
Impact of synchronous or metachronous MPMN **(A)** and interval time **(B)** on patient survival. TF2T, time free to second tumor.

**Figure 4:**
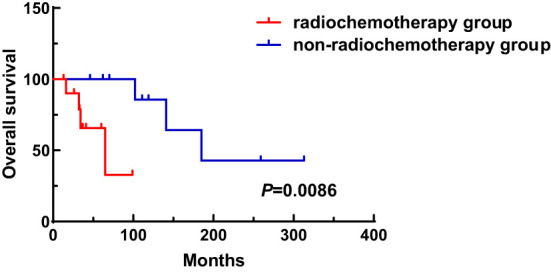
Influence of radiotherapy and chemotherapy on patient survival.

A total of 31 patients were followed-up, with a median follow-up time of 13 months (1–81 months). Overall, 14 patients died as of the follow-up date, resulting in a mortality rate of 45.2% (14/31), including six cases in the synchronous MPMN group and eight cases in the metachronous MPMN group. Factors that may affect patient prognosis in the present study, such as gender, Age 1, Age 2, interval between the occurrence of the two tumors, LDH levels, Ann Arbor staging, IPI score, and B symptoms, were analyzed using Kaplan-Meier and log-rank tests. The results showed no statistically significant difference between males and females (*P*=0.529). For Age 1, the median OS was 185 months for patients <60 years old and 34 months for those ≥60 years old (*P*=0.0013, **Figure 1A**). For Age 2, the median OS was not reached for patients <60 years old, while the median OS was 62 months for those >60 years old (*P*=0.0639, **Figure 1B**). The median OS for the IPI score was not reached in the low/low-middle risk group (0–2 points). It was 16 months for the middle-high/high-risk group (3–5 points; *P*=0.0076, **Figure 2A**). For patients with and without B symptoms, the median OS was 12 months and 141 months, respectively, (*P*=0.0278, **Figure 2B**). The median OS for patients with ECOG≥2 was 62 months, while the median OS for patients with ECOG<2 was not reached (*P*=0.0095, **Figure 2C**). The median OS for patients with elevated LDH levels was 12 months, while those with normal LDH levels had a median OS of 141 months (*P*=0.0019, **Figure 2D**). The median OS for patients with synchronous MPMN was seven months, while for those with metachronous MPMN, it was 141 months (*P*<0.001, **Figure 3A**). The median OS for patients with an interval between the two tumors of <50 months and ≥50 months was 62 months and 185 months, respectively (*P*=0.0273, **Figure 3B**). Except for 10 patients with synchronous tumors, the median survival time for patients with the first primary tumor treated with radiation/chemotherapy and those not treated with radiation/chemotherapy was 65 months and 185 months, respectively (*P*=0.0086, **Figure 4**). Analysis of β_2_-MG levels (*P*=0.105), Ann Arbor stage (*P*=0.083), and Hans classification (*P*=0.604) showed no statistically significant differences. Multivariate analysis using the Cox proportional hazards regression model showed that age of ≥60 years, synchronous tumors, and interval of <50 months between the two tumors were independent prognostic factors affecting patient OS (**Table 5**).

**Table 5:** Multivariate analysis of prognostic factors in DLBCL patients with MPMNs.

Factor	HR(95%CI)	*P* value
Age 1	22.170 (2.133–230.470)	*0.009
IPI	3.683 (0.919–14.762)	0.066
synchronous or metachronous tumors	0.006 (0–0.180)	*0.003
TE2T	0.051 (0.003–0.875)	*0.040

DLBCL, diffuse large B-cell lymphoma; MPMN, multiple primary malignant neoplasm; Age 1, age at first malignancy diagnosis; IPI, International Prognostic Index; TF2T, time free to second tumor; HR, hazard ratio; *P<0.05.

## 4 Discussion

With the improvement of diagnostic and therapeutic techniques in recent years, more and more patients with malignant tumors have the opportunity to achieve long-term survival. However, prolonged survival of patients with the first tumor also increased the incidence of second tumors. Billroth (4) first reported on dual primary tumors over 100 years ago, and subsequent numbers of studies on MPMN have increased. Nevertheless, MPMN pathogenesis remains unclear and is generally believed to be the result of long-term interaction among various carcinogenic factors. Genetic factors, individual susceptibility, and immune status all play key roles in tumor development. Research has shown that there are many risk factors in the development of MPMN in DLBCL, including gene mutations or deletions, genetic susceptibility, familial genetic factors, tumor immunity, and iatrogenic factors, such as the use of radiotherapy and chemotherapy methods (3, 5). Previous studies have considered radiotherapy and chemotherapy to be important risk factors for the occurrence of MPMN, with increased radiation dose and prolonged duration being positively correlated with the risk of MPMN (6, 7). Xu et al. (8) observed that patients with non-Hodgkin lymphoma (NHL) who received a radiation dose of ≥40 Gy had a significantly increased risk of developing lung cancer, breast cancer, and bladder cancer. However, more than half of the patients in the present study received varying degrees of radiotherapy and chemotherapy before developing a second tumor. Whether the occurrence of MPMN is related to this remains uncertain and requires further investigation. Therefore, comprehensive screening should be conducted during a follow-up for these patients to prevent the occurrence of second tumors. Trapani et al. (9) found the immune system to be another important factor. The patient’s immune surveillance and immune defense functions decline when the first primary tumor occurs. Treatment with radiotherapy, chemotherapy, and other measures further damages the immune system, leading to a decrease in immune surveillance, which in turn may trigger the development of MPMN. In future studies, it will be crucial to explore the molecular mechanisms linking DLBCL to secondary tumors. Potential areas of investigation include genetic susceptibility, such as specific mutations or polymorphisms that may predispose patients to secondary malignancies, and immune dysregulation, which could play a significant role in the development of secondary tumors. Understanding these mechanisms could lead to the identification of novel therapeutic targets and improve risk stratification for DLBCL patients.

MPMN is easily confused with metastasis or recurrence of the first tumor, which can delay patient diagnosis and treatment. Repeated pathological biopsies and comprehensive consideration by clinicians are required to confirm the diagnosis. However, there are currently few reports on lymphoma associated with MPMN. Among them, DLBCL as the most common subtype of lymphoma has a reported MPMN incidence of 3.8% in the related literature (10), which was similar to our results. Studies have shown that the proportion of male patients with DLBCL combined with MPMN is significantly higher than that of females (11, 12). In the present study, there were 19 female and only 12 male patients, which is inconsistent with the above studies. This discrepancy may be due to the small sample size and the fact that most of the tumors in women were gynecological cancers. Additionally, metachronous MPMN is more common than synchronous MPMN, with a ratio of 2.1:1. Jiang et al. (13) also reached similar conclusions. When the first tumor is clearly diagnosed, the affected lymph nodes are often considered to be metastases or recurrence, which can lead to misdiagnosis, affecting treatment choice and efficacy evaluation. Involvement of solid organs in lymphoma patients is not always caused by lymphoma invasion. When multiple sites are involved, especially in the case of atypical metastatic lesions, pathological tissue examination is crucial for diagnosing lymphoma and determining its staging. Additionally, not all enlarged lymph nodes are metastases in patients with solid tumors, and lymphoma occurrence should also be considered. In our cohort, there were two patients who were diagnosed with DLBCL and another hematological malignancy concurrently (APL and MDS). As of the latest follow-up, both patients were still alive, with survival durations of 99 months and 10 months, respectively. A study has reported that the incidence of MDS or Acute Myeloid Leukemia as a second malignancy in non-Hodgkin lymphoma patients is 3.8% (14), and these cases are generally associated with poor prognosis. However, due to the very small number of cases involving two hematological malignancies in our study, no meaningful statistical comparison could be performed. Future research with larger sample sizes is needed to validate these findings and to more accurately assess the incidence and prognosis of such cases. A study by Morton et al. (6) showed that the incidence of DLBCL combined with melanoma is significantly increased, but the present study did not identify any melanoma patients. In our research, DLBCL combined with thyroid and lung cancers was the most common, which may be due to racial and regional differences. Further studies with a larger sample size are needed for verification. In China, the incidence of malignant tumors increases with age, and elderly patients have a higher proportion of malignant tumors. In contrast, the median age at the first occurrence of malignant tumors in the present study was <60 years. Thus, more attention should be given to young patients with malignant tumors during diagnosis and follow-up, and appropriate follow-up plans should be developed to monitor for MPMN occurrence.

Currently, there are few studies on the prognosis of DLBCL combined with MPMNs. The present study analyzed factors that may affect patient survival, such as gender, age at onset of malignant tumors, interval between the two tumors, IPI score, B symptoms, LDH levels, and treatment with radiotherapy and chemotherapy. The results showed that gender did not have a statistically significant impact on patient prognosis, which is inconsistent with a study that indicated that female patients have a better prognosis than male patients (15). This discrepancy may be due to the small sample size in the present study, which could lead to a relative error. Future studies with a larger sample size are needed for further investigation.

Many studies have reported that patients with metachronous tumors have a better prognosis than those with synchronous tumors (16, 17). Additionally, Fu et al. (18) found that the longer the interval between the two tumors, the better the prognosis. However, Nishiwaki et al. (19) discovered that the presence of synchronous tumors does not affect patient OS. Therefore, larger sample sizes are still needed to determine the factors influencing patient prognosis. The age of onset of malignant tumors is also an important factor affecting the prognosis of patients with MPMN. In the present study, the median age difference at the onset of the first malignant tumor (~60 years) showed that younger patients had a better prognosis. A study from Tianjin Medical University Cancer Institute and Hospital on 92 cases of NHL with multiple primary malignancies found that age1 of ≥60 years and male gender were unfavorable prognostic factors for patients (13). The present study also found that LDH level above the normal value, IPI score of ≥3, and ECOG score of ≥2 all had adverse effects on patient OS, which was consistent with the findings of another study (20). Zhang et al. (20) also confirmed that patients with non-GCB subtype of DLBCL and MPMN had poorer OS. Moreover, DLBCL patients with MPMN showed shorter OS compared to those with DLBCL alone, indicating that MPMN is associated with a poorer prognosis. This suggests that MPMN may be an independent prognostic factor for DLBCL.However, this result was not observed in the present study. Since our study focused on a retrospective case series of DLBCL patients with MPMN and did not include a control group of DLBCL-only patients, direct statistical comparisons could not be performed. In future DLBCL studies, it is crucial to consider the presence of MPMN. MPMN should be included as a covariate in statistical analyses, or subgroup analyses should be conducted specifically for MPMN patients, to accurately assess its impact. Additionally, given that MPMN subtypes may represent unique biological backgrounds, they should be incorporated into risk stratification models. This will help achieve more precise risk assessment and personalized treatment strategies. Except for the 10 cases of synchronous tumors, the median OS for patients receiving radiotherapy or chemotherapy for the first primary malignancy was different, indicating that these patients had a worse prognosis compared to individuals who did not undergo this treatment, possibly due to factors, such as malignancy of the first primary tumor, pathological staging, radiation/chemotherapy dosage, and overtreatment. In our study, the treatment regimens for DLBCL patients were highly variable, including different combinations of chemotherapeutic agents. Due to individual variability in treatment protocols, the timing and types of drugs used, and the relatively small sample size, it was not possible to determine a clear causal relationship between specific chemotherapeutic agents and the occurrence of secondary tumors in metachronous MPMN patients. Future studies should aim to collect larger cohorts with more standardized treatment protocols to explore potential associations between specific chemotherapeutic agents and the risk of developing secondary malignancies. Additionally, prospective studies incorporating control cohorts of DLBCL patients without MPMN will be essential to validate these findings.

The present study had some limitations. First, it was a single-center retrospective study. Second, the follow-up period was relatively short, the sample size was small, and due to limitations, such as economic conditions, some participants were unable to undergo genetic testing, resulting in missing clinical data, which may have affected the accuracy of the results. Finally, given the limited number of cases, along with the differences in treatment methods and doses for various tumors, it was difficult to perform a more detailed analysis.

In conclusion, the main factors affecting patient prognosis were age of ≥60 years at the time of the first cancer diagnosis and a shorter interval between the two tumors. MPMN needs to be fully recognized in clinical practice and personalized comprehensive treatment plans need to be developed based on the patient’s actual condition and tumor biological characteristics. Comprehensive screening for patients with DLBCL combined with MPMNs should be conducted to avoid misdiagnosis and missed diagnosis. High-risk populations should undergo regular follow-ups, and multidisciplinary collaboration in diagnosis and treatment should be enhanced. Further in-depth exploration of the pathogenesis and characteristics of MPMN is essential to improve its diagnosis and treatment standards.

## Data Availability

The original contributions presented in the study are included in the article/supplementary material. Further inquiries can be directed to the corresponding author.
